# Development of a predictive model for integrated medical and long-term care resource consumption based on health behaviour: application of healthcare big data of patients with circulatory diseases

**DOI:** 10.1186/s12916-020-01874-6

**Published:** 2021-01-08

**Authors:** Tomoyuki Takura, Keiko Hirano Goto, Asao Honda

**Affiliations:** 1 Department of Healthcare Economics and Health Policy, Graduate School of Medicine, The University of Tokyo, 7-3-1 Hongo, Bunkyo-ku, Tokyo, 113-8655 Japan; 2 Department of Cardiovascular Medicine, Juntendo University Faculty of Medicine, Tokyo, Japan; 3 Saitama Inst. of Public Health, Saitama, Japan

**Keywords:** Medical and long-term care resource consumption, Artificial intelligence, Health behaviour, Clinical outcome, Healthcare big data, Circulatory diseases

## Abstract

**Background:**

Medical costs and the burden associated with cardiovascular disease are on the rise. Therefore, to improve the overall economy and quality assessment of the healthcare system, we developed a predictive model of integrated healthcare resource consumption (Adherence Score for Healthcare Resource Outcome, ASHRO) that incorporates patient health behaviours, and examined its association with clinical outcomes.

**Methods:**

This study used information from a large-scale database on health insurance claims, long-term care insurance, and health check-ups. Participants comprised patients who received inpatient medical care for diseases of the circulatory system (ICD-10 codes I00-I99). The predictive model used broadly defined composite adherence as the explanatory variable and medical and long-term care costs as the objective variable. Predictive models used random forest learning (AI: artificial intelligence) to adjust for predictors, and multiple regression analysis to construct ASHRO scores. The ability of discrimination and calibration of the prediction model were evaluated using the area under the curve and the Hosmer-Lemeshow test. We compared the overall mortality of the two ASHRO 50% cut-off groups adjusted for clinical risk factors by propensity score matching over a 48-month follow-up period.

**Results:**

Overall, 48,456 patients were discharged from the hospital with cardiovascular disease (mean age, 68.3 ± 9.9 years; male, 61.9%). The broad adherence score classification, adjusted as an index of the predictive model by machine learning, was an index of eight: secondary prevention, rehabilitation intensity, guidance, proportion of days covered, overlapping outpatient visits/clinical laboratory and physiological tests, medical attendance, and generic drug rate. Multiple regression analysis showed an overall coefficient of determination of 0.313 (*p* < 0.001). Logistic regression analysis with cut-off values of 50% and 25%/75% for medical and long-term care costs showed that the overall coefficient of determination was statistically significant (*p* < 0.001). The score of ASHRO was associated with the incidence of all deaths between the two 50% cut-off groups (2% vs. 7%; *p* < 0.001).

**Conclusions:**

ASHRO accurately predicted future integrated healthcare resource consumption and was associated with clinical outcomes. It can be a valuable tool for evaluating the economic usefulness of individual adherence behaviours and optimising clinical outcomes.

**Supplementary Information:**

The online version contains supplementary material available at 10.1186/s12916-020-01874-6.

## Background

Circulatory diseases are characterised by a wide range of pathological conditions, complicated disease mechanisms, and a tendency to become chronic while repeating acute phase events. These characteristics not only affect the prognosis of life and decrease the patient’s quality of life (QOL), but also place a great burden on society, including the need for long-term care [1, 2]. Although the advancement in drugs and medical devices in this field has been remarkable, the unit cost of medical care has also increased remarkably. In addition, due to changes in lifestyle and other factors, the number of inpatients with cardiovascular disease and other conditions is increasing at a rate of 10,000 per year in Japan [3]. Against this background, medical expenses in the cardiovascular field accounted for 19.7% (in 2018 for patients over 65 years old: an increase of 5.7% from the previous year) of the national medical expenses in Japan, and currently constitute the highest among total medical expenses [4]. As described above, in the cardiovascular field, the effective utilisation of social capital (medical and long-term care expenses) as well as further improvement of clinical results has become a proposition in terms of medical policy [5].

The Ministry of Health, Labour and Welfare reports on medical and long-term care expenses in Japan. Medical costs have exceeded 8% of the gross domestic product (GDP) and have increased by an average of more than 2% annually over the past 10 years [6]. This is due to the ageing population and recent advancements in medical care. Regional differences are mainly caused by frequent and prolonged hospitalisation among the elderly. Meanwhile, long-term care costs have increased by approximately 5% annually since 2010, exceeding 2% of the growth in GDP in Fiscal Year 2016 [7].

It is important to strengthen the management of clinical quality and medical resources. The use of the prediction models for this purpose is also desired. The development of models to predict the severity of patient conditions and the outcome of treatment interventions has been an active part of clinical practice. For example, as models for predicting the vital prognosis of heart failure, comprehensive risk scores such as the Emergency Heart Failure Grade (EHMRG), Multiple Estimation of Risk Based on the Emergency Department Spanish Score in patients with Acute Heart Failure (MEESSI-AHF), Heart Failure Survival Score (HFSS), and Get With the Guidelines–Heart Failure (GWTG-HF) have been developed for acute heart failure, while the Seattle Heart Failure Model (risk stratification) has been developed for chronic heart failure, mainly in Europe and the USA [8, 9]. However, rarely has there been any effort to develop risk assessment and prediction models that contribute to the management of medical and long-term care finance (insurance revenue) on the part of public insurers.

With regard to the management of medical resources, it has become clear that adherence to medication and moral hazards among patients are closely related to clinical outcomes and have a significant impact not only on health behaviours but also on socioeconomic factors, including medical costs [10, 11]. Improvements in medication adherence have been reported to reduce the economic burden as well as the burden of chronic disease on patients [12]. In addition, in the Japanese public healthcare system, which is based on the premise of free access, there is a concern that overlapping examinations, which may lead to adverse events, are common and that the associated increase in medical costs will also be a concern [13]. In addition, disease prevention is an intervention that reduces risk factors and overall costs [14–18]. In contrast, adherence in a broad sense, including self-management and health literacy, may affect disease prevention behaviour and determine its success [19–21]. Moreover, public health policy needs to regulate the fairness of the allocation of healthcare resources in order to avoid the exhaustion of limited shared finances due to random consumption and the deterioration of the well-being and health of the entire population [22, 23]. Thus, it is desirable to develop a model for predicting the consumption of medical resources based on behavioural indicators that reflect adherence and the moral hazards that affect patient choice and behaviour.

However, there are difficulties in collecting information on adherence, and to date, there have been few international empirical studies on this topic. In Japan, there is a large-scale database in which the amount of medical fees claimed in the medical and long-term care fields, the actual structure of overlapping examinations, the record of participation in disease prevention programmes, the results of laboratory tests and biological tests, and information on the prescription rate of generic drugs are unitarily managed for each patient [24, 25]. Therefore, using big data composed of three fields including medical care, long-term care, and health check-ups, we developed a model (Adherence Score for Healthcare Resource Outcome, ASHRO) to predict the cost of medical and long-term care. The prediction model set a composite index of health behaviour including concordance and public nature as broad adherence related to the changes in the clinical and economic burdens in the cardiovascular field, by applying multivariate analysis as well as established machine learning (AI). Thus, ASHRO aims to optimise the collaboration between future patient health behaviours and the healthcare services delivered.

## Method

### Data sources and populations

ASHRO was developed using a national healthcare database (Kokuho Database, KDB) system that links health check-ups, health insurance claims, and long-term care insurance data on an individual basis. The KDB system is a large-scale database which includes self-employed people and retired people who are covered by the National Health Insurance, and the personal collusion rate between medical and long-term care information is more than 99%. The regions included in the database account for 6.1% of the total population of Japan, and in addition to the factors of demographic trends and social structure, the main conditions of medical care and long-term care are generally considered to reflect the average level in Japan. The unified management system for health check-up and medical and long-term care data (KDB) is suitable for predicting medical and long-term care costs, focusing on behavioral changes as a longitudinal study of a wide range.

The prediction model was developed for all ages requiring first to tertiary preventive actions with a history of hospitalisation for cardiovascular diseases (ICD-10 I 00-99) during a 4-year period from April 2014 to March 2018. A single gate was included as a multicentre retrospective observational study of an area with a population of more than 3 million people. Exclusion criteria were as follows: cardiovascular designated intractable diseases on admission, congenital cardiovascular diseases, and serious types of cardiomyopathy.

Each patient’s data would include the following information in the subject’s unified ID after anonymisation processing: basic attributes of the insured person and medical expenses related to hospitalisation, outpatient, dispensing, and dentistry; and information on the diagnosis, breakdown of medical treatment, frequency of medical examinations, period of hospitalisation, cost of long-term care, level of long-term care required, number of times of use, period of use, guidance content of health check-ups, results of laboratory tests and biological tests, and number of times of participation.

This study was approved by the Research Ethics Committee (2018167 N1) of the University of Tokyo (TheBD: Health Economics Big Data, University of Tokyo), and involved strict data confidentiality in accordance with the Helsinki Declaration and the Japanese Government’s Guidelines for Clinical Research Ethics and the REporting of studies Conducted using Observational Routinely-collected Data (RECORD) Statement [26]. This study also complies with the Guidelines for Transparent Reporting of Predictive Models (TORIPOD). The data collected for this study are highly sensitive and, if reasonably requested, can be obtained from the project director.

### Examination of basic model by machine learning

Regarding physical aspects or result completeness, the usual empirical statistical approach—obtain a data set containing results, predictors, and fit coefficients—was not optimal for this exploratory study in terms of performing multivariate analysis on large samples. We therefore chose an approach that exploits machine learning with random forests and *K*-fold cross-validation (Fujitsu Limited Powered by AI) to select and integrate explanatory variables and to set weights. Random forests are machine learning techniques applied to classification and regression. The advantage is that it minimises the problem of overfitting [27, 28]. In medical big data, there is also an advantage that it can be efficiently performed on a large sample with several thousand input variables. In addition, it can support different data scales (for example, blood pressure and GFR have different normal values) and is robust to the inclusion of unrelated variables [29, 30].

In this study, we constructed a basic prediction model for medical and long-term care costs, which is an objective variable, based on machine learning by random forest, by setting more than 100 pieces of information on medical practices, clinical tests, and preventive activities included in big data as explanatory variables. Using this machine learning, we evaluated the integration of parameters and the importance of feature quantities while randomly selecting (bootstrap method: sampling with replacement) multiple sets of samples and feature quantities. The subject data were randomly divided into 80% (training) and 20% (testing) for learning and validation. Mean Decrease Gini (Gini coefficient) was used for classification, and Inc MSE (residual sum of squares: mean square error: out-of-bag [OOB] error rate calculated by fitting the unextracted data to regression trees created by randomly extracted data) was used for regression. As an optimisation measure, a consideration was given to minimising OOB [31, 32]. The number of feature quantities in the generation of the decision tree is the square root of the number of the total variable. The maximum depth of the decision tree was 50.

In this study, a grid search was performed for hyperparameter adjustments in training model generation, and each training model was evaluated by the *K*-fold cross-validation. In addition, the holdout method was used to evaluate the versatility of multiple machine learning algorithms (neural networks) that have already been learned in the above course. The *K*-fold cross-validation, which is a type of internal validation, creates *K* models by rotating test data among *K* groups, and the final prediction value is determined by the average of these models. In this process, OOF (out of fold; prediction) predictions are frequently used. It also randomly determines which samples are placed in which group and sets a parameter called random state.

When aiming for resource management in medical and long-term care, it is important not to fall into the state of “Cheap, but shoddy”. Especially in the public health system, this perspective is an important proposition. Thus, our study aimed to render the prediction model sensitive not only to medical and long-term care costs but also to clinical outcomes. In addition, the setting of the definition of adherence (including moral hazards) in a broad sense and the selection of indicators have an exploratory aspect, and feedback consideration was also necessary. The study also looked at future developments in research, including the expansion of the database and its application to other disease domains. Based on these results, gender, age, and observation period, which are different from the broad adherence index, were added to the basic model using machine learning. From the above, the results of adjusting the classification of non-significant features and similar indicators were reflected in improving the explanatory power of the prediction model and the accuracy of its verification (multivariate analysis: multiple regression analysis, binary logistic regression).

### Outcomes and predictors

In this study, resource consumption (medical and long-term care expenses) was set as an objective variable, and a prediction model was developed using a composite index of adherence in a broad sense as an explanatory variable. Moreover, the current study was analysed from a social point of view. For the objective variable, the costs in this study were the sum of medical costs (hospitalisation, outpatient care, and dispensing) and care costs (home, community-based, and facilities), excluding indirect costs (travel expenses and meals not covered by insurance). The average exchange rate between yen and US dollar was 112.98 yen per US dollar from 2014 to 2017.

As a result of the machine learning, 11 parameters constituting the basic model were selected in the middle from the feature value importance, and finally, they were integrated into 8 parameters. Some of these explanatory variables had a strong relationship with cost (for example: overlapping outer patient visits, medical attendance). In this study, the weighting of each variable was adjusted to improve the explanatory power of the predictive model (Table 1). The main objective of the prediction models examined was to contribute to the proper management of healthcare budget, and because the calculation criteria for each explanatory variable were group averages, it was necessary to consider the characteristics of the target group. Thus, in this study, age, sex, and duration of analysis were further incorporated into the regression analysis as additional explanatory variables, taking into account their scalability to other populations (universality of the model).

**Table 1:** Correlation coefficient of variables and weighting of predictors

Variable	Correlation coefficient	*p* value			Weighting
Age	0.42	<0.001			
**12-month period after enrolment**					
**Health behaviour**				**Broad adherence**	
Secondary prevention			1	Secondary prevention (Integrated)	8.18
Health check-ups	0.122	<0.01		*	
Item of health check-ups	0.128	<0.001		*	
Tertiary prevention					
Rehabilitation intensity	0.012	0.43	2	Rehabilitation intensity	0.81
Guidance	0.156	<0.001	3	Guidance	1.04
PDC	0.079	<0.001	4	PDC	5.35
Overlapping outpatient service					
Outpatient visits	0.001	0.938	5	Overlapping outpatient visits	3.03
Clinical laboratory and physiological tests	0.049	<0.05	6	Overlapping clinical laboratory and physiological test	5.02
Medical attendance			7	Medical attendance (Integrated)	2.76
Inpatient days	0.52	<0.001		**	
Outpatients visits	0.352	<0.001		**	
Dispensing	0.354	<0.001		**	
Public behaviour					
Generic drug rate	0.209	<0.001	8	Generic drug rate	6.58
**Long-term care**					
In-home services, number per year	0.224	<0.001			
Community-based services, number per year	0.011	0.466			
Facility services, number per year	0.086	<0.001			
**48-month follow-up period after index 12-month enrolment**					
Follow-up period	0.442	<0.001			
Medical expense	0.938	<0.001			
Long-term care expense	0.269	<0.001			

Integrated into an adherence 1* or adherence 7** index by machine learning

*Abbreviations*: *PDC* proportion of days covered

The 11 predictors of health behaviour in the final predictive model (ASHRO) were indicators related to health promotion, prevention of disease severity, rational resource consumption behaviour (moral hazard), medical attitude behaviour, and public behaviour (Table 2). The following 11 indicators were calculated as the ratio of the difference between the mean value of the population and the mean of each individual during the 1-year follow-up period after enrolment: number of health check-up/items, units of rehabilitation intensity, number of guidance sessions, number of overlapping outpatient visits/clinical laboratory and physiological tests, inpatient days, number of outpatient visits, dispensing, proportion of days covered (PDC), and generic drug rate. Overlapping outpatient services are similar services for the same disease within the same period. The generic drug rate was the prescription rate with only the original drug based on the generic drug list of the government as the denominator. The PDC was calculated by the duration of the prescription and its continuation, instead of the individual data of actual medication performance.

**Table 2:** Predictor of integrated medical and long-term care resource consumption

Health behaviour		Broad adherence	No.
Secondary prevention	→	Secondary prevention (Integrated)	1
Health check-ups, number per year		*	
Items of check-ups, number per year		*	
Tertiary prevention			
Rehabilitation intensity, units/year	→	Rehabilitation intensity	2
Guidance (e.g. lifestyle-related disease), number per year	→	Guidance	3
PDC, %	→	PDC	4
Overlapping outpatient service			
Outpatients visits, number per year	→	Overlapping outpatient visits	5
Clinical laboratory and physiological tests, number per year	→	Overlapping clinical laboratory and physiological tests	6
Medical attendance behaviour	→	Medical attendance (Integrated)	7
Inpatient days, days per year		**	
Outpatients visits, number per year		**	
Dispensing, number per year		**	
Public behaviour			
Generic drug rate, %	→	Generic drug rate	8
		(Complementary Indicators)	
		Age	
		Sex	
		Follow-up period	

Integrated into an adherence 1* or adherence 7** index by machine learning (see Table 1)

*Abbreviations*: *PDC* proportion of days covered

Not only compliance but also elements of health behaviours such as adherence, moral hazards, and social cooperation (public nature) have been shown to have a significant impact on the consumption of medical resources. The relationship between these explanatory variables, defined as a broad adherence, was preliminarily systematised in this study, focusing on the medical and long-term care costs as the objective variable of the predictive model (Additional file 1). Adherence-related factors (four factors in this study: secondary prevention, rehabilitation intensity, guidance, and PDC) are generally associated with healthcare costs. For example, effects on prescription drug purchase (odds, 1.11) were reported [33]. In addition, the burden of medical expenses for the elderly in Japan is generally 10%, which is lower than that of the working generation at 30%, so it is said that moral hazards are likely to affect them [34, 35]. Factors of moral hazards (in this study, three main factors were considered: overlapping patient visits, overlapping clinical laboratory and physiological tests, and medical attention) have generally been found to affect healthcare costs. For example, in this case, a relationship with general healthcare-seeking behaviour was reported (change in consultation behaviour, 15% decrease) [36]. In addition, elements of social cooperation (in this study, the generic drug rate index was mainly used) have also been found to affect consumption of social security resources. For example, altruistic behaviour toward social welfare was reported in Japan in the evaluation of social cooperation by measuring social value orientation (public nature) [37].

ASHRO is a tool that is expected to be utilised by those in charge of healthcare budget management and specialists in the clinical field. It is characterised by the prediction of medical and long-term care costs by focusing on adherence (behaviour change), which affects clinical and economic indicators. For example, the predictive models were developed for public insurers to conduct various promotions for the ASHRO High groups with low screening and generic drug rates, and to promote guidance by medical professionals in clinical practice. In this context, adherence to primary/secondary prevention is useful for all patients with mild to severe disease and may be linked to studies of substantive behavioural change models that improve self-efficacy, etc., including the development of effective empowerment approaches.

### Statistical analysis and verification method of prediction model

**Fig. 1:**
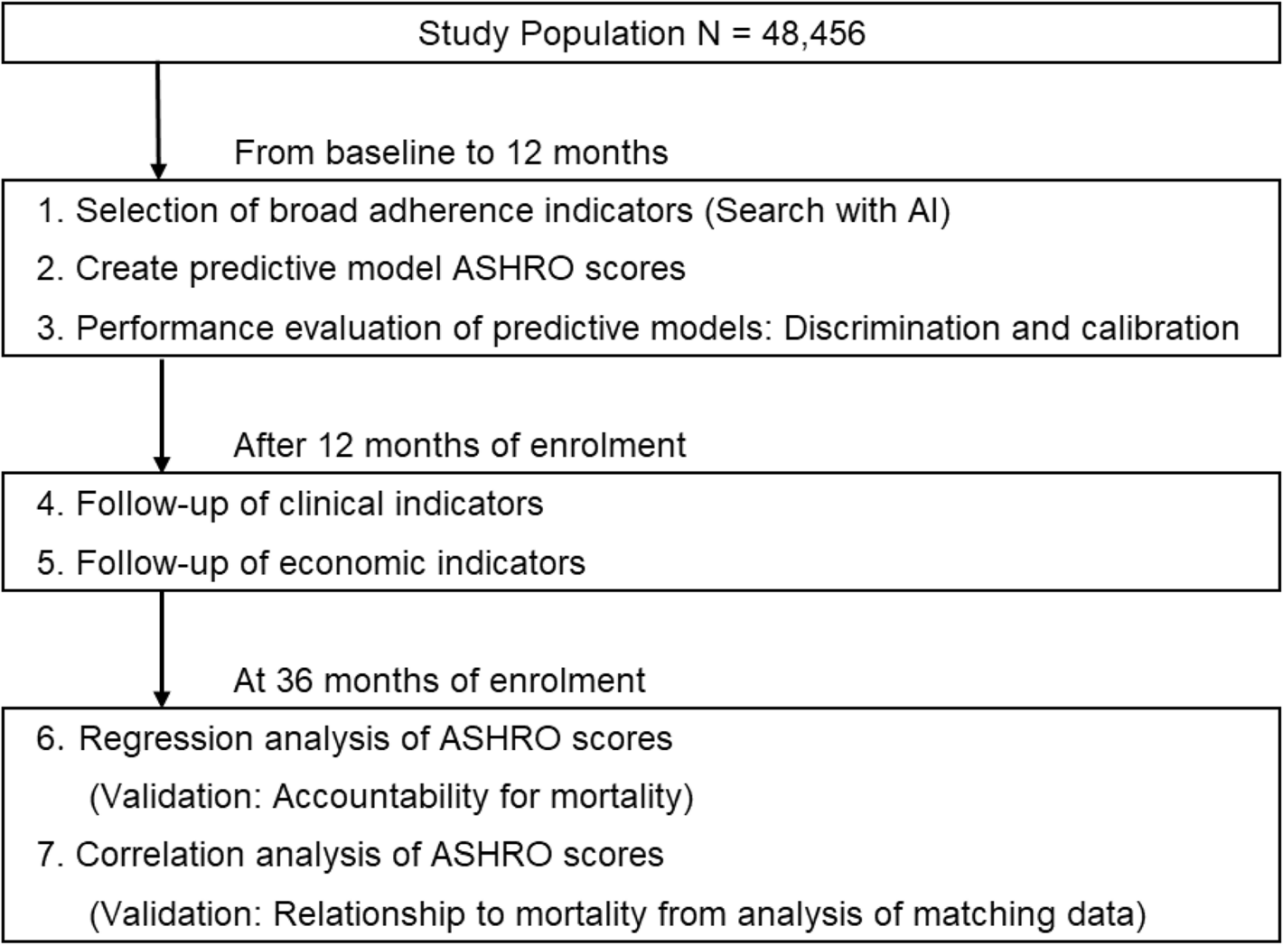
Study flow chart

The objective variables were calculated using data from 12 months after inclusion, and the combined resource consumption of medical and long-term care for the subsequent 36 months was integrated (Fig. 1). For the purpose of improving explanatory power and avoiding multicollinearity, the explanatory variables were selected preferentially for health-related variables, excluding the care-related variables for which a single-correlation analysis was performed. The variation inflation factor (VIF) for verifying multicollinearity did not indicate a problem with the variance of the estimated partial regression coefficient if it was less than approximately 10. Categorical variables were expressed as numeric values (%), and continuous variables were expressed as mean ± standard deviation (SD).

To determine the selection, integration, and weighting of explanatory variables, the aforementioned machine learning and a single-correlation analysis for medical and long-term care costs were performed. A *p* value of up to about 0.5 was allowed for the index selection. In the analysis, the objective variables, medical expenses and long-term care expenses, were divided into 10 categories by rank. Among the explanatory variables, sex, smoking, drinking, and lifestyle modification (posture) were converted into dummy variables.

The prediction model was first built by validation using multiple regression analysis using the forced input method. In a subsequent validation of the prediction model, the sample was divided into two groups, with cut-off values of 50% and 25%/75% to determine medical and long-term care costs, and logistic diffraction was performed. The discrimination and calibration were evaluated using the area under the curve (AUC) and the Hosmer-Lemeshow test.

This predictive model was tested for sensitivity to some clinical outcomes (vital prognosis) and further analysed for correlation with clinical outcomes. Mortality outcomes in relation to clinical outcomes were tested by comparing cumulative overall mortality over a 36-month period between two PS-matched ASHRO 50% cut-offs. We calculated PS for each case as sex, age/BMI (body mass index)/systolic blood pressure/triglycerides/HbA1c/serum creatinine/smoking cessation/alcohol consumption/risk factors and matched the sample size using greedy matching. For reference, we also performed a binary logistic analysis (stepwise method). For other clinical indicators of association with clinical outcomes, a single-correlation analysis confirmed the relationship between ASHRO and the cumulative 36-month differences during follow-up of the health check-up results.

We divided the calculated ASHRO into five stages with the lower limit of ASHRO as 0. For each ASHRO score, the statistics of the amount of variance in medical and long-term care costs relative to the population average were calculated, and the difference in the population mean between each score band was tested. In this study, the statistical significance level was set at 5%. For the machine learning, CARET package version 6.0-86 was used. The software used was SPSS version 26.0 (IBM Corp., Armonk, NY).

## Results

### Baseline characteristics

A total of 48,456 patients were enrolled with an average follow-up period of 36.1 ± 8.8 months. The mean age was 68.3 ± 9.9 years, and the majority of them were male (61.9%). At the baseline major health check-up, BMI was 23.4 ± 3.4 kg/m^2^, systolic blood pressure was 131.2 ± 15.0 mmHg, triglycerides were 20.8 ± 5.2 mg/dL, HbA1c was 5.9% ± 0.8%, and serum creatinine was 0.9 ± 0.8 mg/dL (Table 3). Medical and long-term care costs were 9160 ± 9045 US dollars per year.

**Table 3:** Baseline characteristics of the study population (*n* = 48,456)

Health check-up examination	
Age, years	68.3 ± 9.9
Male sex, *n* (%)	29,994 (61.9)
Physical examination	
Height, cm	160 ± 8.8
Weight, kg	60 ± 11.3
BMI, kg/m^2^	23.4 ± 3.4
Waist, cm	84.4 ± 9.3
Systolic BP, mmHg	131.2 ± 15.0
Diastolic BP, mmHg	75.7 ± 10.3
Lipid profile	
Triglycerides, mg/dL	120.8 ± 75.2
HDL cholesterol, mg/dL	59.4 ± 15.9
LDL cholesterol, mg/dL	116.6 ± 29.3
Kidney function	
Serum creatinine, mg/dL	0.9 ± 0.8
Serum uric acid, mg/dL	5.4 ± 1.4
eGFR, mL/min/1.73m^2^	69.2 ± 17.1
Blood sugar	
HbA1c (%)	5.9 ± 0.8
Follow-up period, months	36.1 ± 8.8

Values are expressed as mean ± SD, *n* (%)

*Abbreviations*: *BP* blood pressure, *BMI* body mass index, *HDL* high-density lipoprotein, *LDL* low-density lipoprotein, *SD* standard deviation

### Selection of predictors

As a result of the machine learning, we integrated the variables and selected 8 parameters related to health behaviour. The weights of the parameters that make up the basic model are as follows: 8.18 for secondary prevention behaviour (integration of number of health check-ups and items), 0.81 for rehabilitation intensity, 1.04 for guidance, 5.35 for PDC classified as tertiary prevention, 3.03 for outpatient visits, 5.02 for clinical laboratory and physical tests classified as overlapping patient service, 2.76 for medical attention (combined with inpatient days, patient visits, and dispensing), and 6.58 for generic drug rate as public behaviour (Table 1).

A single-correlation analysis showed that follow-up period and age were significantly correlated with medical and long-term care costs (*p* < 0.001, Table 1). Of the 11 health behaviour variables before integration, the number of rehabilitation intensity units and the overlapping outpatient visits were not significantly different (*p* = 0.430 and *p* = 0.938, respectively). Other variables were statistically significant (number of clinical laboratory and physiological tests: *p* < 0.05; all others: *p* < 0.001). Many of the care-related variables were also statistically significant.

### Evaluation of prediction models

Multiple regression analysis yielded a coefficient of determination of 0.313 (*p* < 0.001) for the entire model. All eight health behaviour predictors were statistically significant. The standard partial regression coefficients were relatively large: 0.261 for medical attendance, 0.254 for follow-up period, − 0.241 for secondary prevention, and − 0.210 for rehabilitation intensity. Additionally, age was 0.032, and the index of overlapping outpatient visits was 0.053, which was relatively small (Table 4). The stability of the prediction model was confirmed when multicollinearity was verified, and the variance inflation factor (VIF) of all explanatory variables was less than 2.

**Table 4:** Evaluation of prediction models by multiple regression analysis

Index		Partial regression coefficient	Standard partial regression coefficient	F value	*p* value	SE	VIF
Broad adherence							
1	Secondary prevention	-0.048	-0.241	3,820.6	< 0.001	0.001	1.07
2	Rehabilitation intensity	-0.250	-0.210	2,740.7	< 0.001	0.005	1.13
3	Guidance	-0.057	-0.144	1,413.1	< 0.001	0.002	1.03
4	PDC	-0.057	-0.075	366.3	< 0.001	0.003	1.10
5	Overlapping outpatient visits	0.028	0.053	116.4	< 0.001	0.003	1.67
6	Overlapping clinical laboratory and physiological tests	0.012	0.091	343.1	< 0.001	0.001	1.70
7	Medical attendance	0.001	0.261	4,460.5	< 0.001	0.005	1.08
8	Generic drug rate index	-0.019	-0.016	17.7	< 0.001	0.004	1.04
Age		0.009	0.032	56.6	< 0.001	0.001	1.25
Sex		-0.509	-0.086	518.8	< 0.001	0.022	1.01
Follow-up period		0.051	0.254	3,207.9	< 0.001	0.001	1.41
Constant term		3.421		1,249.7	< 0.001	0.105	

*Abbreviations*: *PDC* proportion of days covered, *SE* standard error, *VIF* variance inflation factor

**Fig. 2:**
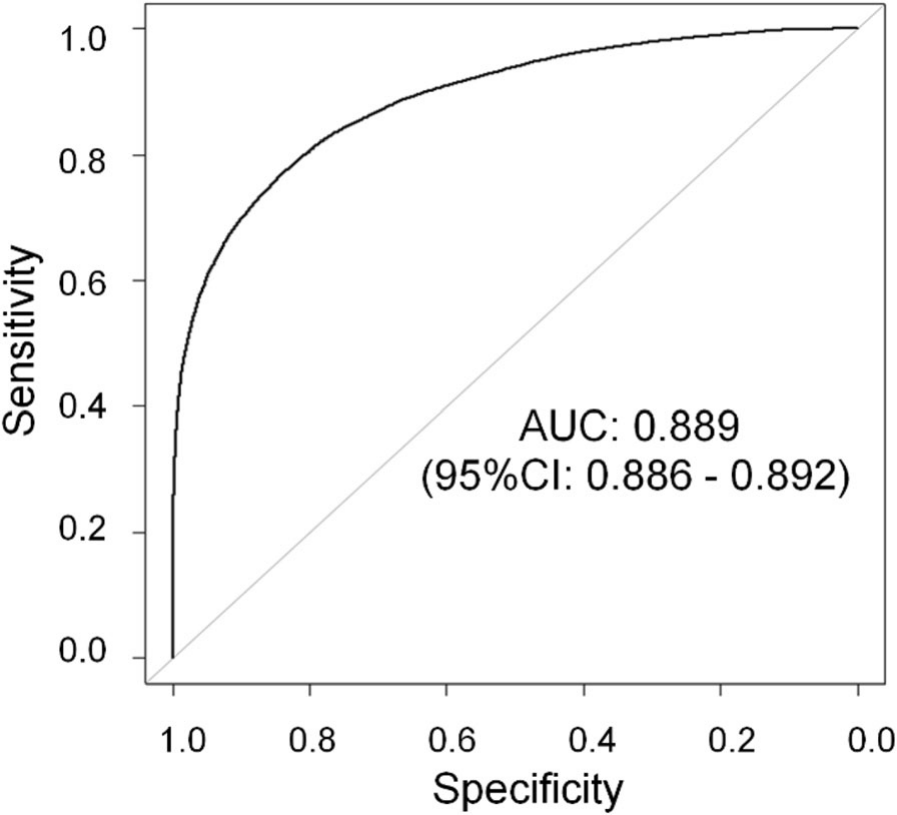
ROC curves of the 36-month prediction model. AUC, area under the curve

As a result of logistic regression analysis for the 50% cut-off of integrated medical and long-term care costs, the coefficient of determination for the entire model was statistically significant (*p* < 0.001, Additional file 2). Other explanatory variables, except for age and overlapping clinical laboratory and physiological tests, showed statistically significant relationships with medical and long-term care costs (all, *p* < 0.001). Many of the partial regression coefficients showed the same tendency as that of the multiple regression analysis. Multicollinearity was confirmed, and the VIF was less than 2 for all explanatory variables. The Hosmer-Lemeshow test was 0.169. The variable with the highest odds ratio was the access index (1.680, 95% CI [confidence interval] 1.660–1.700), and the variable with the lowest odds ratio was the rehabilitation index (0.817, 95% CI 0.804–0.831). The area under the curve (AUC) in the receiver operating characteristic curve (ROC) was 0.889 (95% CI 0.886–0.892, Fig. 2).

Logistic regression analysis with a cut-off of 25%/75% showed that the overall model coefficient of determination was statistically significant (both *p* < 0.001). All VIFs were less than 3 except for a 25% cut-off analysis period (VIF = 2.188). The AUC was 0.880% and 0.896%, respectively (Table 5).

**Table 5:** Logistic regression analysis for the 25%/75% cut-off of integrated medical and long-term care costs

Index		Cut-off	
		Cost: 75%	Cost: 25%
Deviance: overall model			
*p* value		< 0.001	< 0.001
VIF			
Broad adherence score			
1	Secondary prevention	1.0364	1.0906
2	Rehabilitation intensity	1.2010	1.0462
3	Guidance	1.0196	1.0140
4	PDC	1.1190	1.0890
5	Overlapping outpatient visits	1.6915	1.6195
6	Overlapping clinical laboratory and physiological tests	1.7246	1.5868
7	Medical attendance	1.0931	1.6284
8	Generic drug rate index	1.0899	1.0257
Age		1.2097	1.3259
Sex		1.0077	1.0162
Follow-up period		1.4900	2.1885
ROC			
AUC(95% CI)		0.880% (0.876–0.883)	0.896% (0.893–0.899)

*Abbreviations*: *VIF* variance inflation factor, *PDC* proportion of days covered, *ROC* receiver operating characteristic curve, *AUC* area under the ROC curve, *CI* confidence interval

### Association of predictive models with clinical outcomes

In the logistic regression analysis of medical and long-term care costs with a cut-off of 50%, the overall coefficient of determination was as follows: logistic regression of ASHRO scores for mortality indicated odds of 1.860, 1.740–1.980, *p* < 0.001. Propensity score matching for severe risk factors created 6154 patient pairs in the ASHRO 50% cut-off Low and High groups. Both groups had a good balance of age, sex, BMI, systolic blood pressure, triglycerides, HbA1c, serum creatinine, smoking cessation, and alcohol consumption. The 36-month cumulative all-cause mortality in the ASHRO High group was significantly higher than that in the Low group (2% vs. 7%, *p* < 0.001; Table 6). ASHRO was associated with a total 36 months cumulative costs of hospitalisation, outpatient care, and prescription, but not with care costs (Table 7). The average 36 months displacement of the medical examination values was also correlated, except for the long-term care level.

**Table 6:** The 36-month cumulative all-cause mortality rate by ASHRO

Factor	ASHRO matched pair		*p* value
	Low group, *n* = 6154	High group, *n* = 6154	
Male sex, *n* (%)	4307 (70%)	4279 (70%)	0.596
Age, years	69.2 ± 7.1	69.1 ± 6.2	0.397
BMI, kg/m^2^	23.5 ± 3.4	23.5 ± 3.5	0.783
Systolic BP, mmHg	132 ± 15	132 ± 16	0.629
Triglycerides, mg/dL	125 ± 74	125 ± 75	0.965
HbA1c (%)	6.0 ± 0.8	6.0 ± 0.9	0.764
Serum creatinine, mg/dL	0.9 ± 0.8	0.9 ± 0.8	0.490
Smoking (1 = current smoker, 0 = non-smoker)	0.2 ± 0.4	0.2 ± 0.5	0.796
Alcohol drinking, weekly	2.2 ± 0.8	2.2 ± 0.9	0.899
All-cause death, *n* (%)	123 (2%)	430 (7%)	< 0.001

Values in parentheses are standard deviation (SD) and *n* (%)

*Abbreviations*: *ASHRO* Adherence Score for Healthcare Resource Outcome, *BMI* body mass index, *BP* blood pressure

**Table 7:** Correlation between ASHRO and 36-month cumulative costs, and changes in health check-up results

	Correlation coefficient	*p* value
**36 months cumulative costs**		
Medical	0.427	< 0.001
Inpatient	0.373	< 0.001
Outpatient	0.230	< 0.001
Dispensing	0.117	< 0.01
Long-term care	− 0.048	0.288
**Mean displacement for 36 months**		
BMI	0.275	< 0.001
Weight	0.239	< 0.001
Systolic BP	0.299	< 0.001
Triglycerides	0.180	< 0.001
LDL cholesterol	0.279	< 0.001
HbA1c	0.333	< 0.001
Serum uric acid	0.257	< 0.001
Serum creatinine	0.195	< 0.001
eGFR	− 0.285	< 0.001
Level of long-term care required	0.029	0.524
Lifestyle improvement (posture)	− 0.061	0.920
Recommendation for examination (achievements)	0.193	< 0.05

*BMI* body mass index, *BP* blood pressure, *LDL* low-density lipoprotein

### Score and probability distribution of the prediction model

**Fig. 3:**
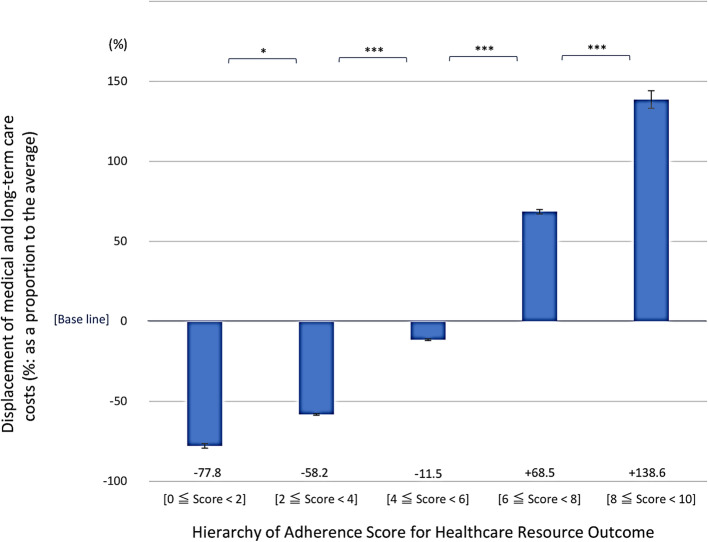
Displacement of medical and long-term care costs by ASHRO score. *p < 0.05, ***p < 0.001. Bar: SE, standard error

The displacements of healthcare integrated resource consumption relative to the population average for each ASHRO score were as follows: the [0 ≦ score < 2] band accounted for − 77.88% (SE 1.48%), the [2 ≦ score < 4] band accounted for − 58.20% (SE 0.45%), the [4 ≦ score < 6] band accounted for − 11.53% (SE 0.51%), the [6 ≦ score < 8] band accounted for 68.51% (SE 1.41%), and the [8 ≦ score < 10] band accounted for 138.65% (SE 5.47%). The maternal mean differences between each score band were statistically significant for all five score bands (score 2: *p* < 0.05, score 2 and above: *p* < 0.001, Fig. 3).

## Discussion

In this study, we applied existing medical data to develop a prediction model that explains resource consumption (medical and long-term care expenses) over 36 months from a composite index of adherence in a broad sense. We chose an approach that exploits machine learning with random forests and *K*-fold cross-validation to select and integrate explanatory variables. In addition to the coefficient of determination of multiple regression analysis, validation of discrimination by AUC and validation of calibration by the Hosmer-Lemeshow test suggested that the developed prediction model (ASHRO) could adequately infer future medical and long-term care costs. Further, ASHRO was also associated with overall mortality in the two 50% cut-off groups adjusted for clinical risk factors.

As the number of comorbidities increases, care becomes more complex, and subsequent clinical outcomes may worsen [38]. However, improved adherence may reduce administrative costs in the consumption of healthcare resources for patients with multimorbidity. The study population—the group of people who have been hospitalised for cardiovascular disease—is often older, and older people who are at risk of multiple chronic diseases and frailty are common. Thus, ASHRO as predictive model was developed in a population that reflects the clinical practice of cardiovascular disease.

**Fig. 4:**
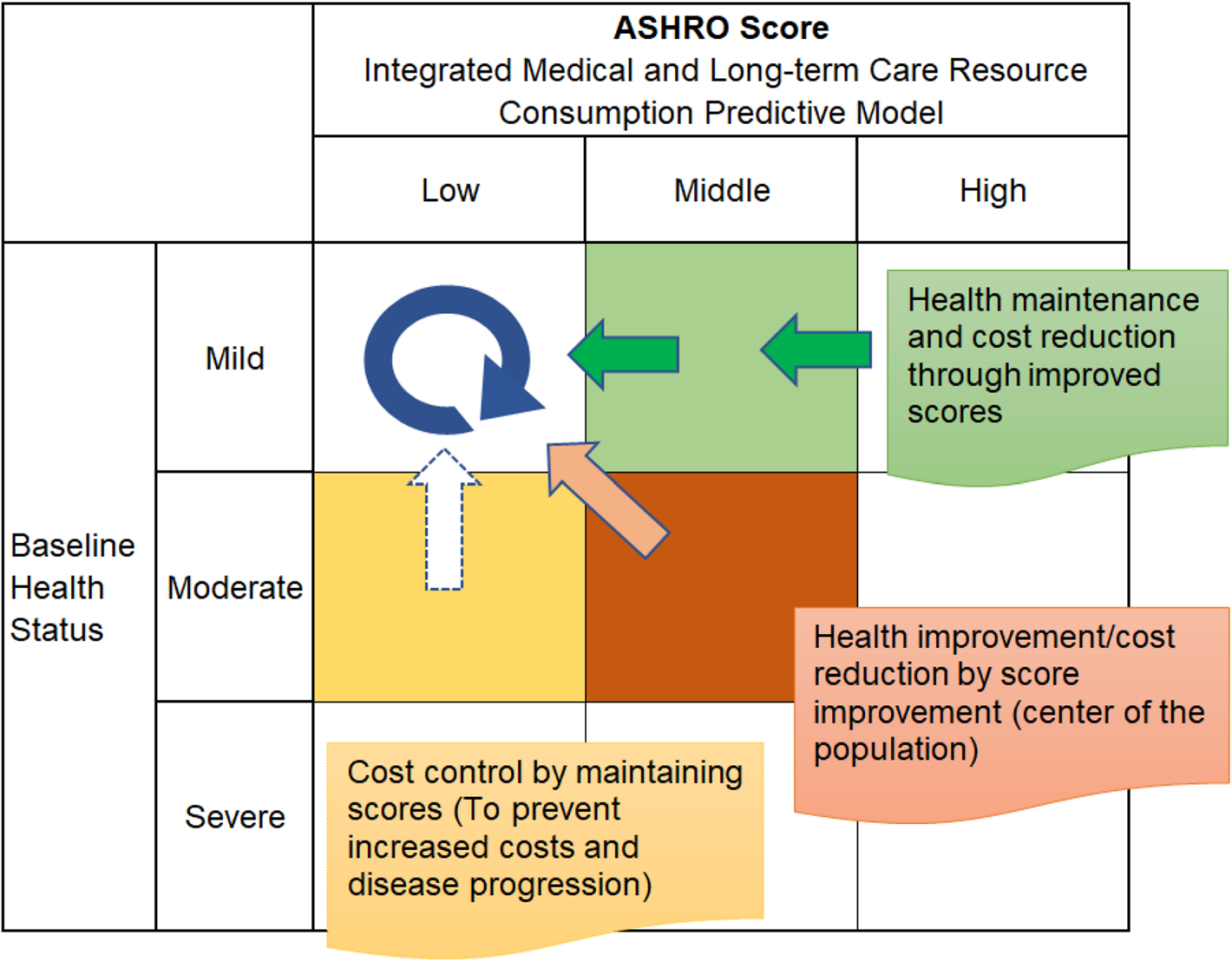
Conceptual diagram of using the calculated ASHRO score. Applying scores in line with the subject’s baseline status will be expected to encourage nudge and behaviour change for the insured and the patient

Predictive models support health behavioural science and health economics research and are used to track the progress of a number of health and health economic indicators. Estimates of available disease management programmes and economic analysis tools, although useful in improving the efficiency of disease management [39], have not been widely standardised among governments, public insurers, and insured parties involved in policy decision-making. ASHRO is a predictive score that is based on adherence rather than clinical indicators and provides new financial and clinical information. The application of medical big data and machine learning (AI) enables the management of behaviour change in large populations. For public insurers, identifying financial risk factors and targets for improvement and sharing them with the insured (nudge) will enable efficient and appropriate financial management of medical and long-term care, leading to the stabilisation of the medical care system (Fig. 4).

Post-acute hospital-to-home healthcare programmes have been suggested to improve medication adherence and clinical indicators, and to reduce rates of adverse cardiovascular events and rehospitalisation [40]. The broader adherence measures of ASHRO predictive models developed for populations with prior hospitalisation for cardiovascular disease may reflect the healthcare provided in transitional care. As the ASHRO score improves, the improvement of clinical outcomes is also expected (Fig. 4; orange). On the other hand, in the case of cerebral infarction, age and disease severity are important factors for predicting the need for long-term care [41]. Although exercise adherence after a typical rehabilitation programme has been described as inadequate [42], it has been reported that high-intensity cardiac rehabilitation is involved in inducing long-term exercise adherence [43]. In order to prevent the ASHRO score from increasing, it is expected that the severity of the disease and prevention of long-term care will be improved in terms of chronic phase medical care (Fig. 4; yellow). In patients with mild disease, the ASHRO score is expected to improve the accuracy of lifestyle modifications and prevent health maintenance and aggravation (Fig. 4; green). Further, the ASHRO predictive model is expected to more actively explain the various factors involved in behavioural change and help improve adherence such as medication.

People with behavioural health concerns have been reported to have a higher burden of care outside of health insurance [44]. Moreover, non-adherence rates of up to 50% have been reported in several studies to date [45–47], and interventions for patients with heart failure have been shown to improve mortality and readmission rates [48], although there is room for improvement [49]. Meanwhile, adherence to more than 80% of the guideline recommendations has been reported to significantly reduce the incidence of major cardiovascular events and reduce costs in post-MI patients [50]. ASHRO, which indicates the degree of adherence to insured persons based on their performance, the level of future cost burden, and goals for improvement in disease risk, is expected to increase self-efficacy, increase awareness of being a member of a social community, and consequently raise the morale of insured persons and improve sensitivity to nudges [51]. As a result, ASHRO is expected to contribute to reducing the burden of local disease and finances.

Adherence indicators in the broad sense are also expected to be linked to studies of substantive behavioural change models that improve self-efficacy. In the future, we plan to analyse the relationship between explanatory variables and conduct model validation for prevention of recurrence of cardiovascular disease, which is a clinical outcome including self-care. Self-efficacy is regarded as a prerequisite for behavioural changes [52] and is correlated with cardiovascular health. However, the optimal timing for incorporating strategies to improve this self-efficacy has not been well established [53]. Moreover, the analysis of explanatory variables is expected to be useful for developing effective empowerment approaches. For instance, regular blood pressure monitoring, positioned as a form of self-care for the management of cardiovascular disease and stroke [54], has been reported to be cost-effective for monitoring interventions in environments where primary care providers and patients work together [55]. Besides, when discussing the behavioural modifications from the aspect of health economics, the balance between economic and clinical aspects is important, as shown in the Zeckhauser dilemma [56]. Therefore, we also verified the sensitivity of the predictive model to vital prognosis.

In Japan, economic trends, such as gross domestic product, are reflected in medical fees, which are closely related to the management activities of medical institutions [57]. In many developed countries, the gap between insurance income and insurance benefits in the social insurance system has become significant against the backdrop of the falling birth rate and ageing population due to changes in population dynamics, the rising cost of medical care due to medical innovations, the increasing complexity of diseases due to the expansion of lifestyle-related diseases, and the low growth due to industrial trends. In Japan, in particular, this divergence started around the year 2000, and the current decline in the balance of payments was on the order of 40 trillion yen [58]. In light of this, it will become increasingly important in the future to harmonise economic and clinical factors in order to continuously develop the medical system represented by the medical insurance system. In this regard, the use of the prediction model developed in this study is highly significant.

### Limitations

Machine learning with random forests has several disadvantages. First of all, there is no absolute criterion for the importance of the feature quantity, and there are cases in which it is difficult to decide what level is important. In addition, due to the existence of randomness in the approach, the importance of the feature quantity fluctuates with each learning, and the selection and integration of parameters may be restricted. Furthermore, the generalisation performance of complex data is lower than that of classification methods such as support vector machines (SVM). In addition, in light of the purpose of the development of the prediction model developed in this research, it is conceivable that data will be updated over time, and the prediction accuracy and model structure will be constantly modified. In such cases, the use of machine learning, including random forests, may still be limited in terms of efficiency and versatility. Furthermore, it is desirable to verify the results of model development study by calibration incorporating split-sample validation cross-validation, and bootstrap validation. It should be noted that although this cross-validation is useful when it is difficult to add specimens, there is a concern that it may lead to overfitting. In the future, we plan to further improve such internal validation methods while considering the characteristics of each method. In view of the above, it is desirable to further investigate the validity of the overall AI approach after examining the developed model based on many aspects.

Next, we did not perform highly reliable external validation (no validation on an independent data set) in evaluating the fitness of the developed prediction model, and we consider this to be one of the limitations of this research. When evaluating the goodness of fit of a logistic regression model or the like, likelihood ratio test statistics, contribution rates, discriminant accuracy, Hosmer-Lemeshow test, etc. are used. The Hosmer-Lemeshow test is used in many large-scale database studies because of its ease of implementation and simplicity of interpretation. It is a method used to evaluate whether the distribution of “actual observed frequency” and “frequency predicted from the model” as a whole is different by comprehensively evaluating the difference between observed frequency and expected frequency in each subgroup. When the sample size is very large, the Hosmer-Lemeshow statistic can yield a false-positive result [59]. On the other hand, if the sample size is less than approximately 500, the test may fail to detect models with low power and poor calibration. Furthermore, the number of risk groups that are chosen will influence the outcome. Generally, it is often set to 10, but the rationale for determining the exact number of groups is not sufficient. It is not necessarily consistent with other measures of conformance (likelihood ratios, contribution rates, positive discrimination rates, etc.). Thus, in interpreting the results obtained in this study, attention should be paid to the possibility of false positives in the context of large sample sizes.

Moreover, in using ASHRO, it is essential to improve or maintain clinical outcomes, rather than simply reduce healthcare costs. Among the selected predictors, there were measurement limitations for adherence factors (the four components of the study were secondary prevention, rehabilitation intensity, guidance, and PDC). Measurements do not include health behaviours for early detection of diseases other than specific health check-ups for all insured persons between the ages of 40 and 74, or self-care implemented as a personal strategy other than dietary guidance and rehabilitation provided. Although the standard reference values for the predictors in this study are the arithmetic mean of the population mean, it is also possible that, based on a more pathological background of the disease, the model may become more versatile if the standard values are a drug adherence level that can actually improve clinical outcomes or a rehabilitation intensity that induces long-term exercise adherence.

This study established behavioural indicators as broad adherence to health behaviours from the performance of patient and healthcare provider behaviour choices in health check-ups, health insurance claims, and long-term care insurance databases. In the future, it is necessary to examine the background of actual action and its significance in detail. For example, the position of the second opinion in duplicate practices, the limited selection for the clinical characteristic in the generic drug rate, and the clinical need or severity in the access frequency were mentioned. In this study, the concept of adherence was expanded and applied in view of trends in the awareness and understanding of moral hazards and concordance in clinical practice; therefore, the definition of active involvement of patients may be controversial in the future [60–63].

Due to the use of real-world data, the developed ASHRO score generally ranged from 0 to 10, but a very small number (0.3%) of non-convergent samples tended to be distributed over a score of 10. This was an expected result for us, but it included a certain error in associating these scores with medical and long-term care costs in five stages. It is possible to examine the possibility of sorting out the points of generating a certain error by logarithmic processing and odds ratios in the future; however, at present, we assumed that there is no problem in treating the change ratio to the population average as a risk fluctuation.

The developed prediction model assumes mutual interference and a trade-off between the medical and long-term care fields and sets the objective variable as integrated medical and long-term care resource consumption. However, considering the decrease in prediction accuracy and improvement in appearance due to multicollinearity and overfitting in multiple regression analysis, we did not include behavioural indicators related to long-term care in the predictors. Therefore, the model’s sensitivity to long-term care costs was low. In the future, it is also desirable to study a model that is sensitive to long-term care.

## Conclusion

ASHRO enables the assessment of the economic value of individual adherence behaviours and may accelerate health behaviours in cardiovascular disease management strategies. This study is the first to evaluate the optimisation of resource consumption and clinical outcomes by health behaviours. The results obtained are likely to contribute to the sustainable development of the medical system.

### Lay summary

A) The predictive model (ASHRO) calculates a score that predicts healthcare resource consumption (10 scales; see the “Score and probability distribution of the prediction model” section).
B) The explanatory variables, which are predictors, can be preliminarily explained as a theoretical framework that focuses on medical and long-term care costs, which are objective variables. The integrated parameter, which was named a broad adherence in this study, is complemented by moral hazards and social cooperation (public nature). These broadly defined adherence indicators may be collaborated to studies of substantive behavioural change models that improve self-efficacy, etc. (see the “Outcomes and predictors” section).
C) ASHRO predictive scores are generated using the national healthcare database (Kokuho Database, KDB) system, which links health check-ups, health insurance claims, and long-term care insurance data on an individual basis. The explanatory variables for adherence in the broad sense are selected and adjusted using AI. The final prediction model is organised and evaluated by multiple regression analysis (see the “Data sources and populations” section).
D) ASHRO is a tool that is expected to be utilised by those in charge of healthcare budget management and specialists in the clinical field. Predictive models enable the insured to share the level of future cost burden and the goal of reducing disease risk with the public insurer and medical professionals (see the “Discussion” section).
E) Analysis of explanatory variables is expected to be used by public insurers in conducting various promotions for health check-ups and high scoring groups with low generic drug rates, and in promoting guidance provided by medical professionals (see the “Outcomes and predictors” section).
F) The predictive models have significant limitations that have not been validated by external data. The relationships between variables have not been verified and must be verified in the future. Further research is needed to link this research with research on a practical behavioural modification model that improves self-efficacy (see the “Limitations” section).

## Supplementary Information

**Additional file 1: Figure S1.** Theoretical framework for explanatory variables focusing on medical and long-term care costs (.doc file). The relationship between the explanatory variables that make up a broad adherence can be preliminarily explained as a theoretical framework by focusing on medical and long-term care costs, which are objective variables of the prediction model. The background to the above is that the predictors were searched and selected as explanatory variables for resource consumption.

**Additional file 2: Table S1.** Logistic regression analysis for 50% cut-off of integrated medical and long-term care costs.

## Data Availability

Data collected for this study are highly sensitive, and if reasonably requested, data supporting the findings of this study can be obtained from the project director.

## Abbreviations

ASHRO: Adherence Score for Healthcare Resource Outcome
AI: Artificial intelligence
PDC: Proportion of days covered
BMI: Body mass index
AUC: Area under the curve
VIF: Variance inflation factor
