# Supplementary figures and images for: Development of a predictive model for integrated medical and long-term care resource consumption based on health behaviour: application of healthcare big data of patients with circulatory diseases
Source: BMC Med. 2021 Jan 8;19:15. doi: 10.1186/s12916-020-01874-6 (PMC7792071; doi:10.1186/s12916-020-01874-6)

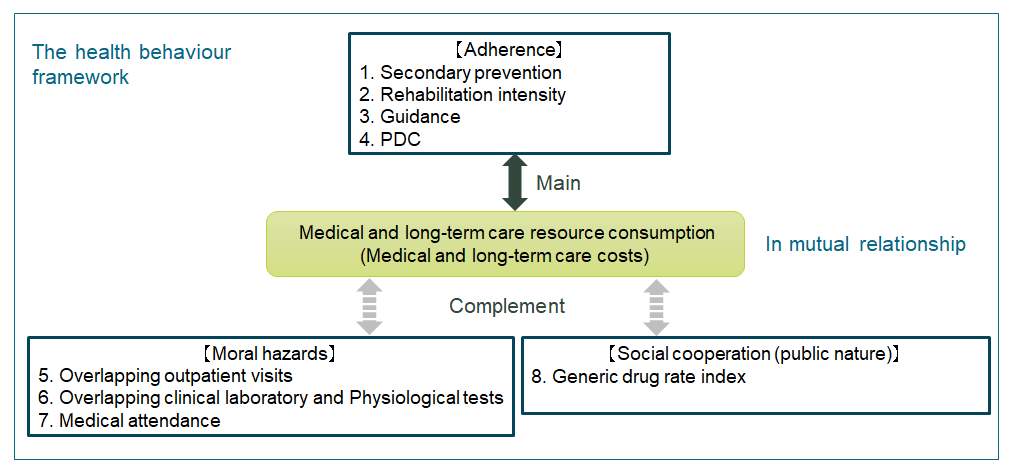

Supplement: Supplementary file 1 — Additional file 1: Figure S1. Theoretical framework for explanatory variables focusing on medical and long-term care costs (.doc file). The relationship between the explanatory variables that make up a broad adherence can be preliminarily explained as a theoretical framework by focusing on medical and long-term care costs, which are objective variables of the prediction model. The background to the above is that the predictors were searched and selected as explanatory variables for resource consumption. [file 12916_2020_1874_MOESM1_ESM.docx]
