# Supplementary material for: Development of a predictive model for integrated medical and long-term care resource consumption based on health behaviour: application of healthcare big data of patients with circulatory diseases
Source: BMC Med. 2021 Jan 8;19:15. doi: 10.1186/s12916-020-01874-6 (PMC7792071; doi:10.1186/s12916-020-01874-6)
Supplement: Supplementary file 2 — Additional file 2: Table S1. Logistic regression analysis for 50% cut-off of integrated medical and long-term care costs. [file 12916_2020_1874_MOESM2_ESM.docx]

**Table S1. Logistic regression analysis for 50% cut-off of integrated medical and long-term care costs**

|  |  | Coefficient | | | | | Odds | |
| --- | --- | --- | --- | --- | --- | --- | --- | --- |
| Index | | Estimate | z value | p value | SE | VIF | Odds ratio | 95%CI |
| Broad adherence score | |  |  |  |  |  |  |  |
| 1 | Secondary prevention | -0.028 | -31.3 | < 0.001 | 0.001 | 1.070 | 0.972 | (0.970 − 0.974) |
| 2 | Rehabilitation intensity | -0.202 | -24.1 | < 0.001 | 0.008 | 1.123 | 0.817 | (0.804 − 0.831) |
| 3 | Guidance | -0.053 | -22.4 | < 0.001 | 0.002 | 1.018 | 0.949 | (0.944 − 0.953) |
| 4 | PDC | -0.057 | -19.2 | < 0.001 | 0.003 | 1.113 | 0.944 | (0.939 − 0.950) |
| 5 | Overlapping outpatient visits | -0.002 | -0.8 | 0.450 | 0.003 | 1.629 | 0.998 | (0.992 −1.000) |
| 6 | Overlapping clinical laboratory and physiological tests | 0.004 | 5.5 | < 0.001 | 0.001 | 1.640 | 1.000 | (1.000 − 1.010) |
| 7 | Medical attendance | 0.519 | 77.7 | < 0.001 | 0.007 | 1.350 | 1.680 | (1.660 −1.700) |
| 8 | Generic drug rate index | -0.065 | -13.2 | < 0.001 | 0.005 | 1.053 | 0.937 | (0.928 − 0.946) |
| Age | | -0.003 | -1.9 | 0.060 | 0.001 | 1.285 | 0.997 | (0.995 − 1.000) |
| Sex | | -0.297 | -11.6 | < 0.001 | 0.026 | 1.008 | 0.743 | (0.707 −0.781) |
| Follow-up period | | -0.006 | -5.2 | < 0.001 | 0.001 | 1.891 | 0.994 | (0.992 − 0.996) |
| Constant term | | 3.574 | 25.6 | < 0.001 | 0.140 |  |  |  |
| Deviance: Overall Model | |  |  | < 0.001 |  |  |  |  |
| Hosmer-Lemeshow test | |  |  | 0.169 |  |  |  |  |

Abbreviations: PDC, proportion of days covered; SE, Standard error; VIF, variance inflation factor; CI, confidence interval
